# Expression and Functional Analysis of lncRNAs Involved in Platelet-Derived Growth Factor-BB-Induced Proliferation of Human Aortic Smooth Muscle Cells

**DOI:** 10.3389/fcvm.2021.702718

**Published:** 2021-09-07

**Authors:** Jia-Jie Lin, Wei Chen, Miao Gong, Xin Xu, Mei-Yang Du, Si-Fan Wang, Li-Yun Yang, Yu Wang, Ke-Xin Liu, Peng Kong, Bin Li, Kun Liu, Yi-Ming Li, Li-Hua Dong, Shao-Guang Sun

**Affiliations:** ^1^Department of Biochemistry and Molecular Biology, Key Laboratory of Medical Biotechnology of Hebei Province, Cardiovascular Medical Science Center, Hebei Medical University, Shijiazhuang, China; ^2^Stem Cell Translational Research Center, Tongji Hospital, Tongji University School of Medicine, Shanghai, China

**Keywords:** long non-coding RNA, human aortic smooth muscle cell, proliferation, HIF1A-AS2, PDGF

## Abstract

Abnormal proliferation of vascular smooth muscle cells (VSMCs) is a common feature of many vascular remodeling diseases. Because long non-coding RNAs (lncRNAs) play a critical role in cardiovascular diseases, we analyzed the key lncRNAs that regulate VSMC proliferation. Microarray analysis identified 2,643 differentially expressed lncRNAs (DELs) and 3,720 differentially expressed coding genes (DEGs) between fetal bovine serum (FBS) starvation-induced quiescent human aortic smooth muscle cells (HASMCs) and platelet-derived growth factor-BB (PDGF-BB)-stimulated proliferative HASMCs. Gene Ontology and pathway analyses of the identified DEGs and DELs demonstrated that many lncRNAs were enriched in pathways related to cell proliferation. One of the upregulated lncRNAs in proliferative HASMC was HIF1A anti-sense RNA 2 (HIF1A-AS2). HIF1A-AS2 suppression decreased HASMC proliferation via the miR-30e-5p/CCND2 mRNA axis. We have thus identified key DELs and DEGs involved in the regulation of PDGF-BB induced HASMC proliferation. Moreover, HIF1A-AS2 promotes HASMC proliferation, suggesting its potential involvement in VSMC proliferative vascular diseases.

## Introduction

Vascular smooth muscle cells (VSMCs), the main cells that constitute blood vessels, play a critical role in maintaining their normal physiological function (1). Abnormal proliferation of VSMCs is a common feature of many vascular remodeling diseases, including atherosclerosis (2), hypertension (3), and vascular aneurysms (4). Thus, regulation of VSMC proliferation has major implications for the prevention of pathological vascular conditions (5).

Long non-coding RNAs (lncRNAs) function in the regulation of gene expression by recruiting chromatin-remodeling complexes, acting as competing endogenous RNAs (ceRNAs) to sponge microRNAs (miRNAs), interacting with transcripts or RNA binding proteins (RBPs), and regulating RNA splicing/editing/transport (6). LncRNAs have emerged as critical regulators of various VSMC functions, including proliferation, migration, and apoptosis; they act as ceRNAs to sponge miRNAs in VSMCs (7–10). For example, by binding miR-148b, lncRNA H19 upregulates Wnt family member 1 (WNT1), thereby facilitating proliferation and inhibiting apoptosis of VSMCs (7). LncRNA MEG3 sponges miR-361-5p to upregulate expression of ATP-binding cassette transporter A1 (ABCA1), which inhibits proliferation and induces apoptosis of VSMCs (8). LncRNA GAS5 binds to miR-21, thus relieving inhibition of programmed cell death 4 (PDCD4) and suppressing platelet-derived growth factor-BB (PDGF-BB)-induced VSMC proliferation and migration (9). LncRNA C2dat1 promotes expression of sirtuin 1 (SIRT1) by targeting miR-34a-5p, thus enhancing VSMC proliferation and migration (10).

In addition, lncRNAs bind to RBPs in VSMCs (11–13). LincRNA-p21 binds to mouse double minute 2 (MDM2), thus enhancing p53 activity and inhibiting VSMC proliferation (11). Binding of lncRNA NEAT1 to WD repeat domain 5 (WDR5) sequesters the latter from the smooth muscle (SM)-specific gene loci, thus downregulating expression of SM-specific genes and promoting VSMC proliferation (12). LncRNA AK098656 binds to myosin heavy chain 11 (MYH11) and fibronectin 1 (FN1), resulting in their degradation and thus increasing VSMC proliferation and migration (13).

Vascular injury induces the release of platelet-derived growth factor (PDGF) by activated inflammatory cells, platelets, and VSMCs, resulting in a switch from VSMC differentiated phenotype to a proliferative phenotype (14–16). However, lncRNAs involved in human aortic smooth muscle cell (HASMC) proliferation activated by the PDGF-BB isoform remain obscure.

In this study, using microarrays, we analyzed differentially expressed lncRNAs (DELs) and differentially expressed coding genes (DEGs) in proliferative HASMCs induced by PDGF-BB and quiescent HASMCs induced by fetal bovine serum (FBS) starvation. Our data showed that many lncRNAs were enriched in Gene Ontology (GO) terms and pathways related to cell proliferation. In addition, knockdown of HIF1A antisense RNA 2 (HIF1A-AS2) inhibited HASMC proliferation, at least in part, via the miR-30e-5p/CCND2 mRNA axis. Our study provides vital clues for elucidating the lncRNAs exert in VSMC abnormal proliferation, as it relates to VSMC proliferative vascular diseases.

## Materials and Methods

### Cell Culture

Human aortic smooth muscle cells (HASMCs, ScienCell, California, USA) were cultured in Smooth Muscle Cell Medium (SMCM, ScienCell) containing 2% FBS, 1% smooth muscle cell growth supplement (SMCGS), and 1% penicillin/streptomycin solution in a humidified atmosphere containing 5% CO_2_ at 37°C. Quiescent HASMCs were induced by FBS and SMCGS starvation for 24 h, proliferative HASMCs were obtained from quiescent HASMCs after 24 h treatment with 10 ng/mL PDGF-BB (R&D Systems Inc., Minneapolis, USA).

### RNA Extraction and Hybridization

Total RNA was isolated from proliferative and quiescent HASMCs using TRIzol reagent (Life Technologies, Carlsbad, USA). Quantity and quality of RNA were measured by NanoDrop ND-1000 (Thermo Fisher Scientific). RNA integrity was assessed by standard denaturing agarose gel electrophoresis. Arraystar Human LncRNA Microarray V3.0 was performed for detection of lncRNA and mRNA expression; 30,586 lncRNAs and 26,109 coding genes could be detected. Sample labeling and array hybridization were performed according to the Agilent One-Color Microarray-Based Gene Expression Analysis protocol (Agilent Technology, California, USA). Briefly, mRNA was purified from total RNA after removal of rRNA (mRNA-ONLY™ Eukaryotic mRNA Isolation Kit, Epicenter Technologies, Illinois, USA). Each sample was amplified and transcribed into fluorescent cRNA along the entire length of the transcripts without 3′ bias utilizing a random priming method (Quick Amp Labeling Kit, One-Color, Agilent Technology). Labeled cRNAs were purified by RNeasy Mini Kit (QIAGEN, Dusseldorf, Germany). Concentration and specific activity of the labeled cRNAs (pmol Cy3/μg cRNA) were measured by NanoDrop ND-1000; 1 μg of each labeled cRNA was fragmented by adding 5 μL of 10 × Blocking Agent and 1 μL of 25 × Fragmentation Buffer and then heated at 60°C for 30 min. Finally, 25 μL of 2 × GE Hybridization Buffer was added to dilute the labeled cRNA; 50 μL of hybridization solution was dispensed into the gasket slide and assembled to the lncRNA expression microarray slide. Slides were incubated for 17 h at 65°C in an Agilent Hybridization Oven. Hybridized arrays were washed, fixed, and scanned using the Agilent DNA Microarray Scanner. Microarray analysis was performed by Kangchen Bio-tech, Shanghai, China. Microarray data described in this paper have been deposited in NCBI Gene Expression Omnibus (GEO) and are accessible with the GEO Series accession number GSE77279 (https://www.ncbi.nlm.nih.gov/geo/query/acc.cgi?acc=GSE77279).

### Quantitative Real-Time PCR (qRT-PCR)

Total RNA was isolated and quantified as described above. Total RNA was treated with DNase I (Takara, Dalian, China) to remove genomic DNA and reversely transcribed using M-MLV First Strand Kit (Thermo Fisher Scientific). qRT-PCR was performed using FastStart Universal SYBR Green Master Mix (Roche, Basel, Switzerland). Reaction conditions were as follows: a denaturation step of 5 min at 93°C, followed by 40 cycles of 30 s at 93°C and 30 s at 52°C, and a final step of 15 s at 72°C. All samples were normalized to internal control β-actin, and the 2^−ΔΔCt^ method was used to calculate relative fold changes. Primers were listed in Supplementary Table 1. The experiment was repeated three times for each gene.

### GO and Pathway Analyses

GO analysis was a functional analysis associating DEGs with GO categories. GO categories were derived from Gene Ontology (www.geneontology.org) and divided into biological processes (BP), cellular components (CC), and molecular functions (MF). Pathway analysis is an effective method to uncover the underlying biological functions in response to DEGs. Based on the latest Kyoto Encyclopedia of Genes and Genomes (http://www.genome.jp/kegg) database, we performed pathway analysis for DEGs. *p* < 0.05 was the threshold for statistical significance.

### Construction of DELs-DEGs Co-expression Networks and DELs-miRNAs Interaction Networks

Pearson correlation coefficient (PCC) was calculated; *R*-value was utilized to calculate the PCC correlation coefficient between six DELs and DEGs from microarray data. Based on PCC (using the selection parameter PCC ≥ 0.90 as meaningful), the co-expression networks were constructed using Cytoscape_v3.7.1. In addition, we selected six DELs with AGO2 binding regions, predicted miRNAs interactions with these DELs using DIANA-LncBase (17), and constructed interaction networks utilizing Cytoscape_v3.7.1.

### SiRNA Transfection

SiRNA-HIF1A-AS2 and siRNA-Control were designed and purchased from GenePharma, China. Sequence of siRNA-HIF1A-AS2 was: 5′-CAGGAAACUUAAGCUUACATT-3′ and 5′-UGUAAGCUUAAGUUUCCUGTT-3′. Sequence of siRNA-Control was: 5′-UUCUCCGAACGUGUCACGUTT-3′ and 5′-ACGUGACACGUUCGGAGAATT-3′. SiRNAs and HiperFect transfection reagent were diluted in a serum-free medium and mixed (30 min, RT) in an equal-ratio to form a polyplex, according to the manufacturer's instructions (HiperFect Transfection Kit; QIAGEN). Polyplex mixture was then incubated 6 h with HASMCs in a humidified incubator containing 95% air and 5% CO_2_ at 37°C. After transfection, polyplex mixture was replaced with fresh SMCM containing 10 ng/mL PDGF-BB (R&D Systems).

### Western Blot Analysis

Proteins were extracted using RIPA buffer (Solarbio, Beijing, China) containing 1 mM phenylmethylsulfonyl fluoride (Solarbio). Protein concentration was determined using the Bradford method (Solarbio). Equal amounts of proteins were separated by SDS-PAGE and transferred onto a PVDF membrane (Merck, Germany). After blocking with 5% non-fat milk in TBST for 2 h, the membranes were incubated with primary antibodies against CCND2 (1:500, Proteintech, Wuhan, China), PCNA (1:1,000, Proteintech), Ki-67 (1:500, Wanleibio, Shenyang, China), p-ERK1/2 (1:300, Wanleibio), p-p38 (1:750, Wanleibio), p-JNK (1:300, Wanleibio), MMP9 (1:1,000, Wanleibio), MMP2 (1:500, Wanleibio), α-SMA (1:500, Wanleibio), GAPDH (1:1,000, Proteintech) and β-tubulin (1:1,000, Proteintech) at 4°C overnight. After washing with TBST, the membranes were then incubated with goat anti-rabbit IgG secondary antibody (dilution at a 1:20,000, Wanleibio) at 37°C for 2 h. Signal was visualized by ChemiDoc^TM^ MP Imaging System (BIO-RAD, California, USA). The experiment was repeated at least three times.

### Cell Viability Assay

Cell viability was measured using CCK-8 assay kit (Wanleibio). In brief, HASMCs transfected with control or HIF1A-AS2 siRNA were seeded into 96-well plates and cultured in a medium containing 10 ng/mL PDGF-BB for 48 h. HASMCs were incubated with 10% CCK-8 solution 2 h in a humidified incubator containing 95% air and 5% CO_2_ at 37°C, and absorbance was measured at 450 nm. The experiment was repeated three times.

### EdU Incorporation Assay

Incorporation assay of 5-Ethynyl-2′-deoxyuridine (EdU) was performed using BeyoClick™ EdU Cell Proliferation Kit with Alexa Fluor 594 (Beyotime, Shanghai, China). HASMCs were seeded in 24-well plates at a density of 2 × 10^5^ cells/well, and each sample contained 6 duplicate wells. SiRNA-Control or siRNA-HIF1A-AS2 were transfected into HASMCs and cultured with or without 10 ng/mL PDGF-BB. After 48 h of siRNA treatment, each well was incubated with SMCM containing 10 μM EdU for 2 h. HASMCs were fixed by 100 μL 4% paraformaldehyde for 15 min. After washing with PBS, HASMCs were incubated with 100 μL PBS containing 0.3% TritonX-100 for 15 min. After washing with PBS, HASMCs were stained with 100 μL Click Additive Solution for 30 min. After washing with PBS, nuclear staining was performed using 1 × Hoechst 33,342 incubation for 30 min. After washing with PBS, the positive cells were observed by fluorescence microscope (OLYMPUS IX71). The experiment was repeated three times.

### Nuclear and Cytoplasmic RNA Detection

Nuclear/cytoplasmic components of proliferative HASMCs induced by 10 ng/mL PDGF-BB were isolated utilizing the Nuclear/Cytosol Fractionation Kit (BioVision, California, USA) according to the manufacturer's instructions. Extraction, quantification, and integrity detection of RNAs were the same as above. Reaction condition of qRT-PCR was in line with the above. U6 and GAPDH acted as positive controls for the cytoplasm and nucleus, respectively. Primers were exhibited in Supplementary Table 1. The experiment was repeated three times.

### Dual-Luciferase Reporter Gene Assay

RNAhybrid was performed to analyze the potential binding sites between HIF1A-AS2 or CCND2 and miR-30e-5p. Using GP-transfect-Mate (GenePharma), luciferase reporter gene vectors (psi-CHECK^TM^-2 Vector, Promega, USA) containing wild-type (WT) or mutant (MUT) HIF1A-AS2 and the 3′-UTR of WT or MUT CCND2 were co-transfected into 293A cells with miR-30e-5p mimics or miR-Control (GenePharma). After 24 h, Dual-Luciferase® Reporter Assay System (Promega) was performed based on the manufacturer's instructions. For each analysis, the Renilla luciferase signal was normalized to the firefly luciferase signal. The experiment was repeated three times.

### Statistical Analysis

Data presented as bar graphs are the means ± SEM or SD of at least three independent experiments. Statistical analysis between two groups was performed using the Student's *t*-test. Differences between multiple groups were assessed by one-way ANOVA with Tukey's multiple comparisons test. Value of *p* < 0.05 was considered statistically significance.

## Results

### Identification of DELs Between Proliferative and Quiescent HASMCs

**Figure 1:**
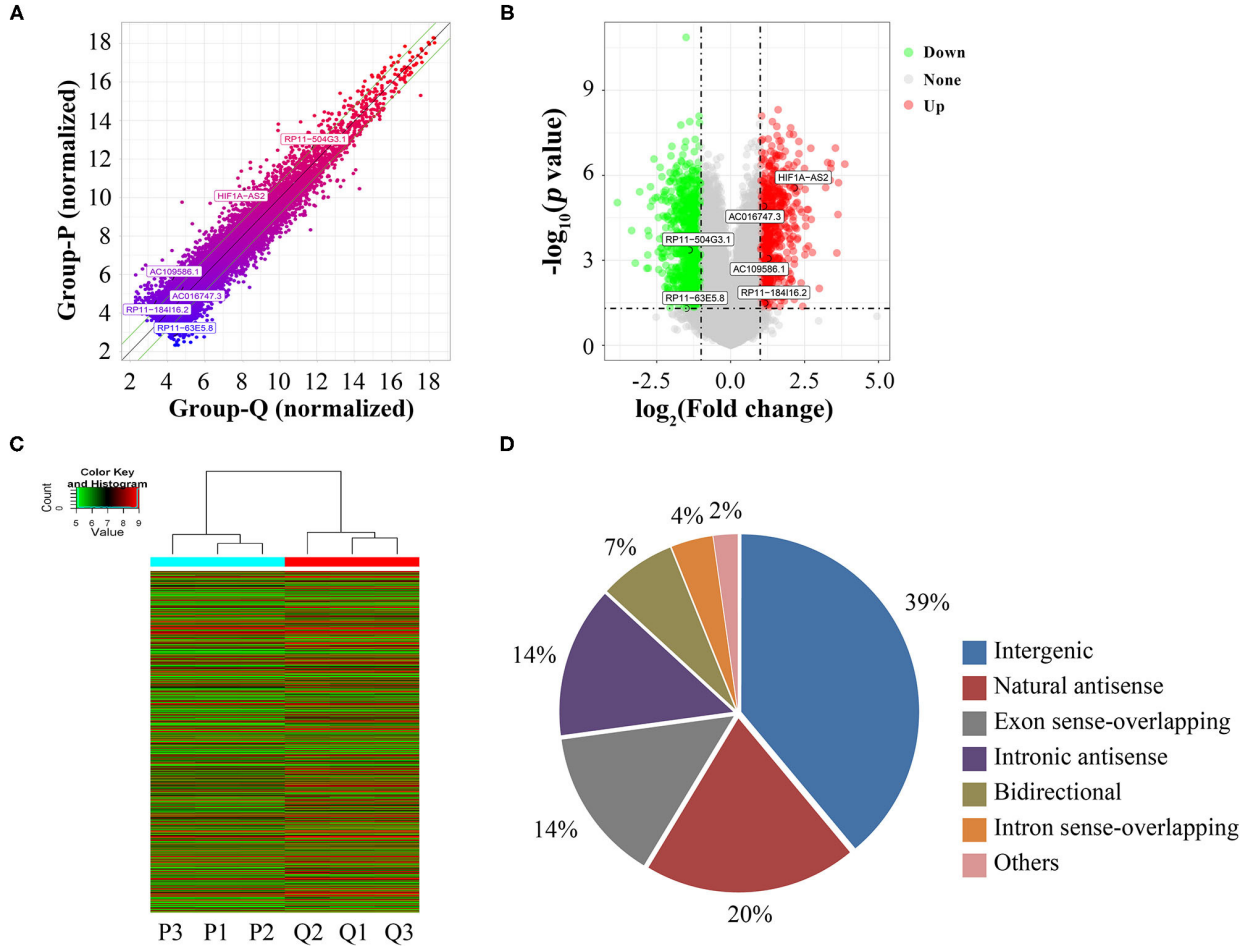
Identification of DELs between proliferative and quiescent HASMCs. **(A)** Scatter plot of lncRNA expression in proliferative and quiescent HASMCs. Values of X and Y axes in the scatter plots are normalized signal values of the samples (log_2_ scaled). Green lines are fold-change lines, with the default fold change value being 2, lncRNAs above the top green line and below the bottom green line with more than 2-fold changes between proliferative and quiescent HASMCs. **(B)** Volcano plot of DELs between proliferative and quiescent HASMCs. Vertical lines correspond to 2-fold differences; horizontal line represents a *p*-value of 0.05. Red and green points represent DELs with statistical significance. **(C)** Hierarchical clustering was performed to show differential lncRNA expression profiling in proliferative and quiescent HASMCs. Cluster analysis arranged samples into groups based on their expression levels; ‘red’ denotes high relative expression, and ‘green’ denotes low relative expression. **(D)** Categories and distributions of DELs. P, Proliferative HASMCs; Q, Qui-escent HASMCs. P1, P2, and P3 indicated three samples of proliferative HASMCs; Q1, Q2, and Q3 indicated three samples of quiescent HASMCs.

Using microarrays, we screened 30,586 lncRNAs to investigate their expression profiles in proliferative and quiescent HASMCs. Scatter plot (Figure 1A), volcano plot (Figure 1B), and heatmap (Figure 1C) showed that compared with quiescent HASMCs, proliferative HASMCs contained 1,095 up-regulated DELs and 1,548 down-regulated DELs (fold change ≥2, *p* < 0.05). DELs (Supplementary Table 2) included 1,029 intergenic lncRNAs, 521 natural sense lncRNAs, 375 exon sense-overlapping lncRNAs, 370 intronic antisense lncRNAs, 187 bidirectional lncRNAs, 102 intron sense-overlapping lncRNAs, and 59 other lncRNAs (Figure 1D).

### PCR Validation of Microarray Data

**Figure 2:**
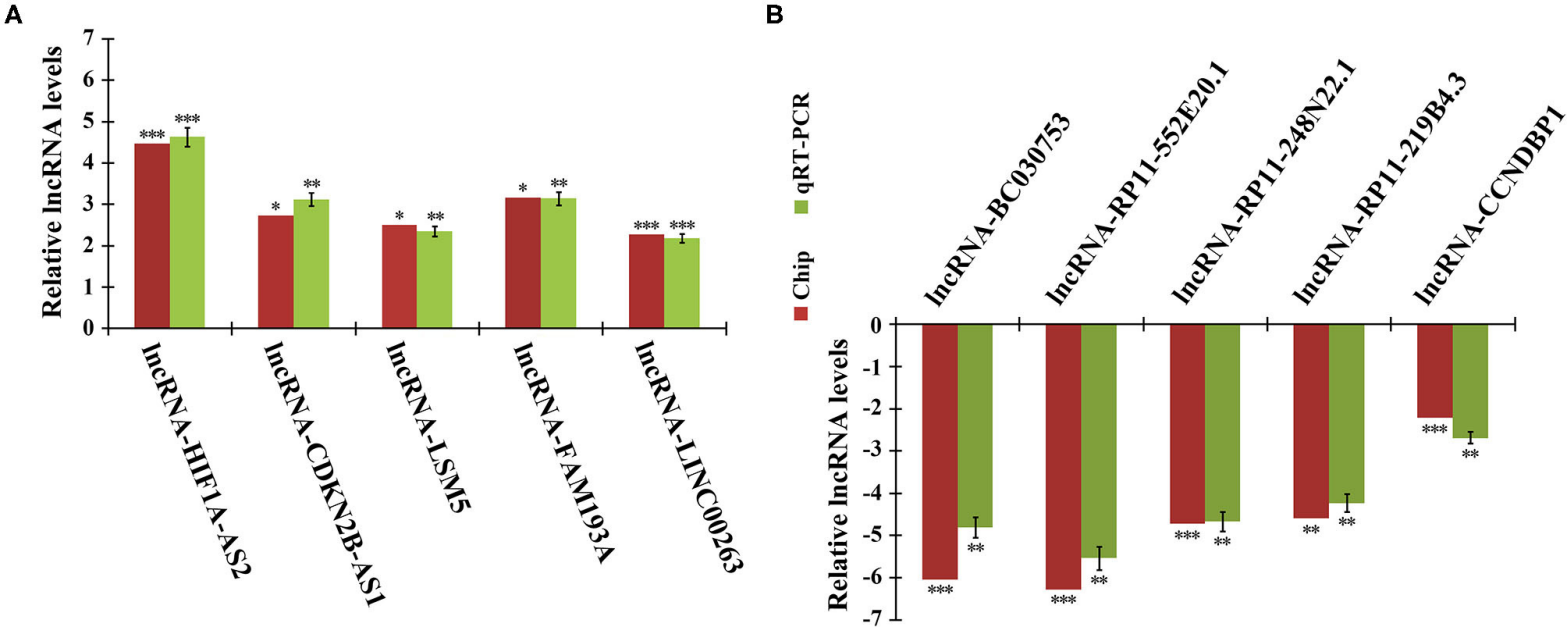
qRT-PCR validation of microarray data. Expression of 10 DELs in proliferative and quiescent HASMCs was validated by qRT-PCR and normalized to β-actin. **(A)** Five up-regulated lncRNAs: HIF1A-AS2, CDKN2B-AS1, LSM5, FAM193A, and LINC00263. **(B)** Five down-regulated lncRNAs: BC030753, RP11-552E20.1, RP11-248N22.1, RP11-219B4.3, and CCNDBP1. Data are presented as mean ± SEM of three independent experiments, *p < 0.05, **p < 0.01, ***p < 0.001 vs. quiescent HASMCs group.

To validate the reliability of the microarray data, we randomly selected 10 DELs for quantitative real-time PCR (qRT-PCR) analysis. In agreement with microarray, compared to quiescent HASMCs, lncRNA HIF1A-AS2, CDKN2B-AS1, LSM5, FAM193A, and LINC00263 were up-regulated, whereas BC030753, RP11-552E20.1, RP11-248N22.1, RP11-219B4.3, and CCNDBP1 were down-regulated in proliferative HASMCs (Figure 2).

### Identification of DEGs Between Proliferative and Quiescent HASMCs

**Figure 3:**
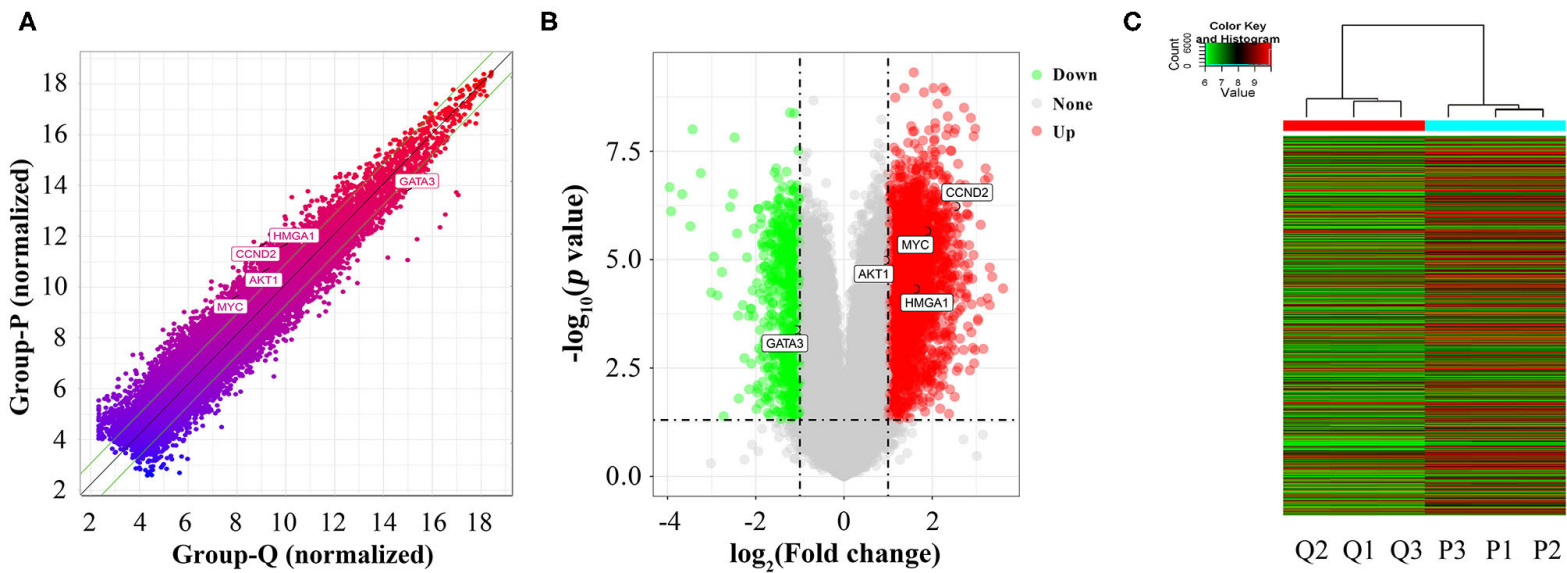
Identification of DEGs between proliferative and quiescent HASMCs. **(A)** Scatter plot of DEGs between proliferative and quiescent HASMCs. **(B)** Volcano plot of DEGs between proliferative and quiescent HASMCs. **(C)** Hierarchical clustering was performed to show differential coding gene expression profiling in proliferative and quiescent HASMCs. P, Proliferative HASMCs; Q, Quiescent HASMCs. P1, P2, and P3 indicated three samples of proliferative HASMCs; Q1, Q2, and Q3 indicated three samples of quiescent HASMCs.

A total of 26,109 coding genes were analyzed by microarrays in HASMCs; from these, 3,720 DEGs in proliferative and quiescent HASMCs were identified (fold change ≥2, *p* < 0.05). DEGs (Supplementary Table 3) included 3,019 up-regulated and 701 down-regulated DEGs (Figure 3).

### GO and Pathway Analyses of DEGs

**Figure 4:**
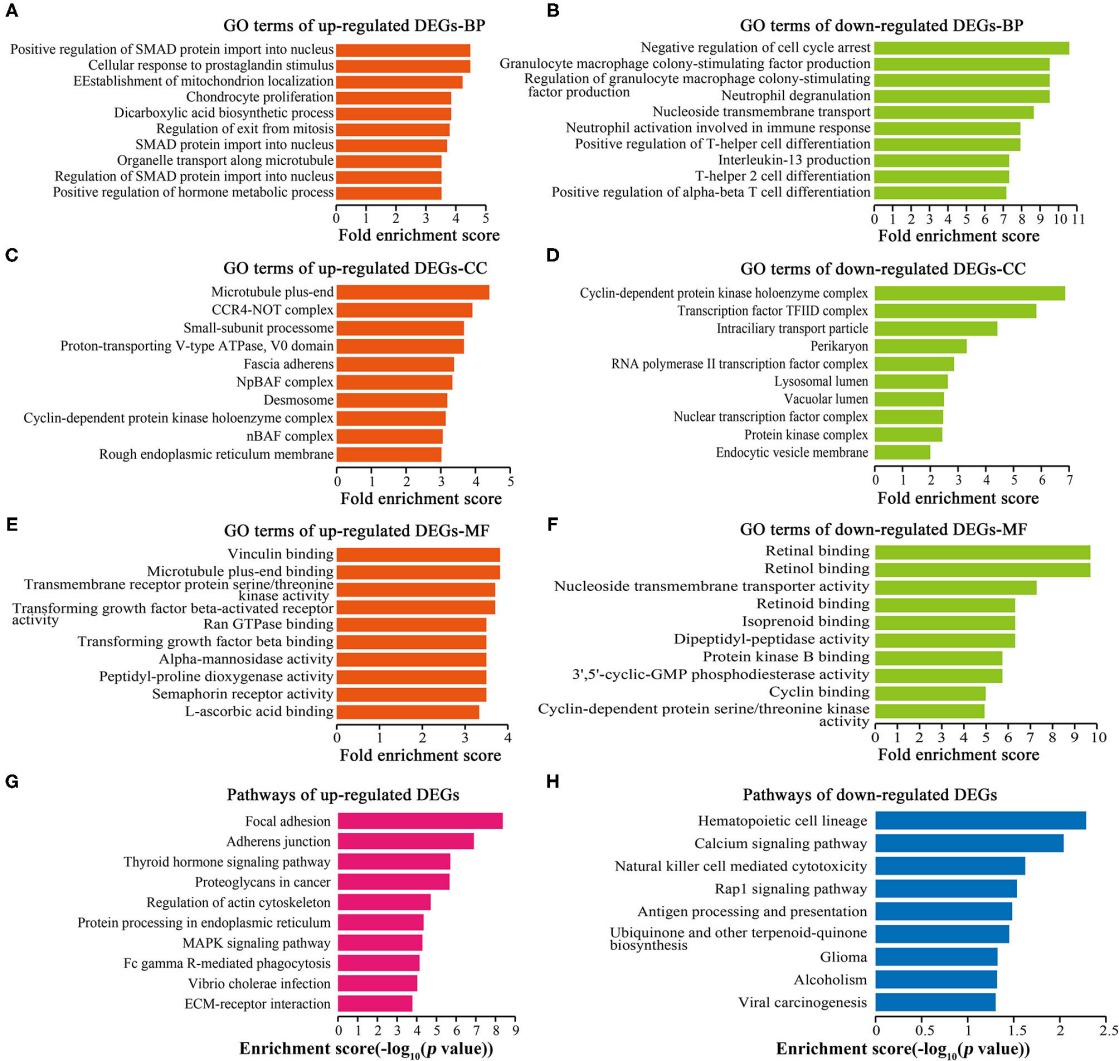
GO and pathway analyses of DEGs. Top 10 GO terms ranked by fold enrichment score are shown in **(A)** BP terms, **(C)** CC terms, **(E)** MF terms for the up-regulated DEGs; **(B)** BP terms, **(D)** CC terms, and **(F)** MF terms for the down-regulated DEGs. **(G)** Top 10 enrichment pathways of up-regulated DEGs. **(H)** Top 10 enrichment pathways of down-regulated DEGs.

GO analysis showed that 1,449 up-regulated DEGs and 196 down-regulated DEGs were enriched in biological processes (BP) (Figures 4A,B), 259 up-regulated DEGs and 14 down-regulated DEGs were enriched in cellular components (CC) (Figures 4C,D), and 254 up-regulated DEGs and 41 down-regulated DEGs were enriched in molecular functions (MF) (Figures 4E,F). Cell proliferation-related GO terms were significantly enriched, such as small mother against decapentaplegic (SMAD) associated-BP (Figure 4A), negative regulation of cell arrest associated-BP (Figure 4B), cyclin-dependent protein kinase associated-CC (Figures 4C,D), transforming growth factor-beta (TGF-beta) associated-MF (Figure 4E), and cyclin-dependent protein kinase associated-MF (Figure 4F). Cell proliferation-related pathways were enriched in the regulation of actin cytoskeleton (Figure 4G), MAPK signaling pathway (Figure 4G), and calcium signaling pathway (Figure 4H).

### GO and Pathway Analyses of DEGs Neighboring DELs

**Figure 5:**
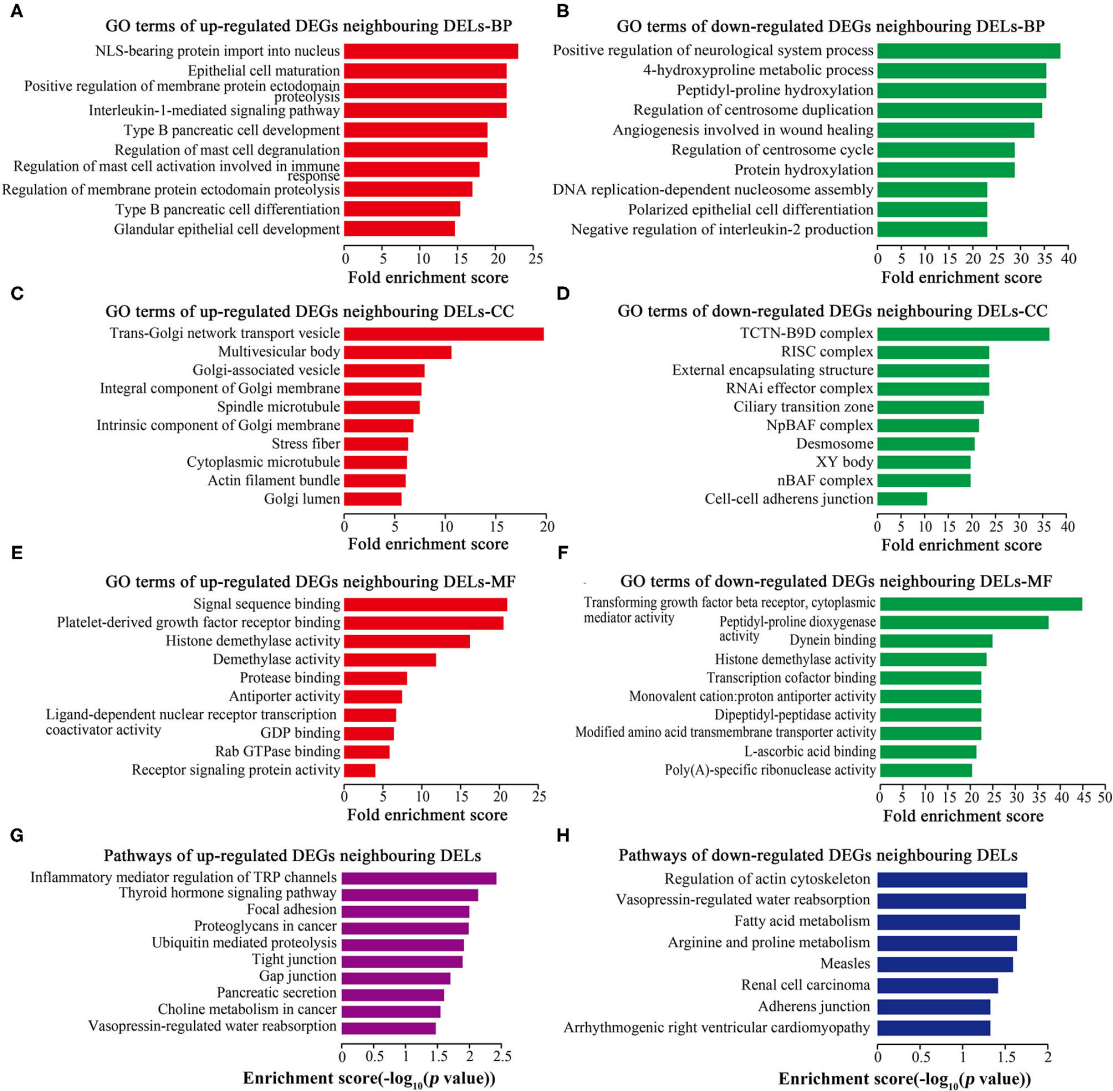
GO and pathway analyses of DEGs neighboring DELs. Top 10 GO terms ranked by fold enrichment score are shown in **(A)** BP terms, **(C)** CC terms, and **(E)** MF terms for neighboring DEGs of up-regulated DELs; **(B)** BP terms, **(D)** CC terms, and **(F)** MF terms for neighboring DEGs of down-regulated DELs. **(G)** Top 10 enrichment pathways of neighboring DEGs of up-regulated DELs. **(H)** Top 10 enrichment pathways of neighboring DEGs of down-regulated DELs.

GO analysis showed that 251 up-regulated neighboring DEGs and 260 down-regulated neighboring DEGs were enriched in BP (Figures 5A,B), 64 up-regulated neighboring DEGs and 63 down-regulated neighboring DEGs were enriched in CC (Figures 5C,D), and 27 up-regulated neighboring DEGs and 48 down-regulated neighboring DEGs were enriched in MF (Figures 5E,F). Cell proliferation-related GO terms, such as RNA-induced silencing complex (RISC) associated-CC (Figure 5D), PDGF receptor binding associated-MF (Figure 5E), and TGF-beta receptor binding associated-MF (Figure 5F) were significantly enriched. Pathway analysis (Figures 5G,H) revealed some cell proliferation-related pathways, such as the regulation of actin cytoskeleton.

### Construction of DELs-miRNAs Interaction Networks

**Figure 6:**
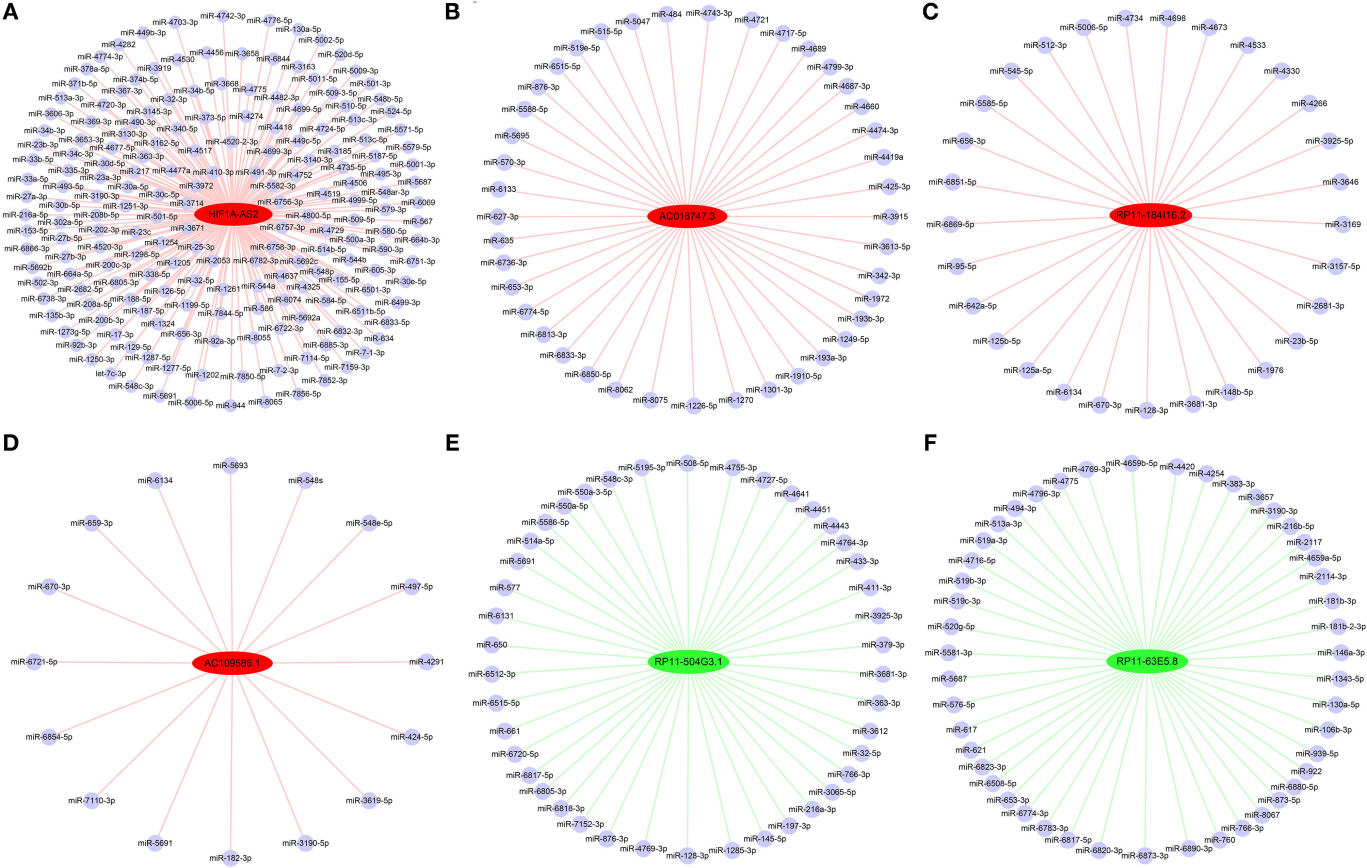
Interaction networks between DELs and miRNAs in HASMCs. Subnetworks of **(A)** HIF1A-AS2, **(B)** AC016747.3, **(C)** RP11-184I16.2, **(D)** AC109586.1, **(E)** RP11-504G3.1, and **(F)** RP11-63E5.8. Red nodes: up-regulated DELs; green nodes: down-regulated DELs; light blue nodes: miRNAs that might interact with DELs.

From the identified DELs, we analyzed 33 DELs containing Argonaute 2 (AGO2) binding sites, so these lncRNAs might function as miRNA sponges. Using DIANA-LncBase (17), we predicted miRNA binding sites in four upregulated DELs (HIF1A-AS2, AC016747.3, RP11-184I16.2, and AC109586.1) and two downregulated DELs (RP11-504G3.1 and RP11-63E5.8). Construction of DELs-miRNAs interaction networks (Figure 6), indicated that HIF1A-AS2 might sponge 187 miRNAs, including miR-30e-5p (18–20), miR-25-3p (21), miR-200c-3p (5), miR-490-3p (22), and miR-34b-5p (23); these five miRNAs are demonstrated to suppress VSMC proliferation (Figure 6A). AC016747.3 might sponge 41 miRNAs, including miR-342-3p (24) that has been shown to inhibit VSMC proliferation (Figure 6B). RP11-184I16.2 might sponge 29 miRNAs including miR-128-3p (25) that inhibits VSMC proliferation (Figure 6C). AC109586.1 might sponge 16 miRNAs, including two miRNAs [miR-182-3p (26) and miR-424-5p (27)] that have been confirmed to inhibit VSMC proliferation (Figure 6D). In addition, RP11-504G3.1 might sponge 42 miRNAs, including miR-433-3p (28) that promotes VSMC proliferation (Figure 6E). RP11-63E5.8 might sponge 47 miRNAs, including VSMC proliferation promoter miR-494-3p (28) (Figure 6F).

### Construction of DELs-DEGs Co-expression Networks

**Figure 7:**
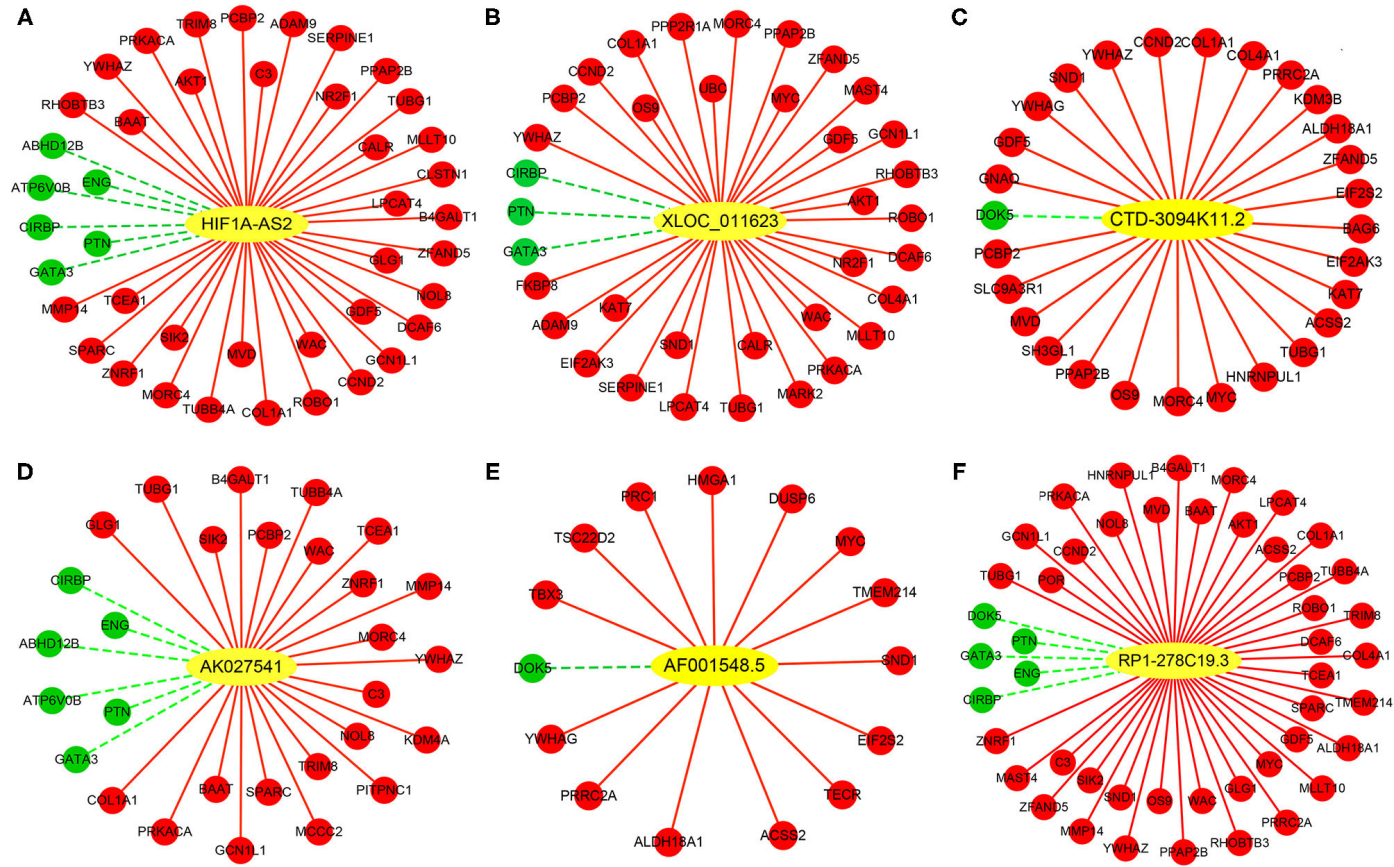
Co-expression networks between DELs and DEGs in HASMCs. Subnetworks of **(A)** HIF1A-AS2, **(B)** XLOC_011623, **(C)** CTD-3094K11.2, **(D)** AK027541, **(E)** AF001548.5, and **(F)** RP1-278C19.3. Yellow nodes indicate up-regulated DELs, red nodes indicate up-regulated DEGs, and green nodes indicate down-regulated DEGs.

Co-expression networks were constructed based on 6 DELs and their co-expression DEGs, including HIF1A-AS2, XLOC_011623, CTD-3094K11.2, AK027541, AF001548.5, and RP1-278C19.3 (Figure 7). From these networks, we found that several important VSMC proliferation-associated DEGs were co-expressed with the lncRNAs (29–33). HIF1A-AS2 was co-expressed with 42 DEGs, including cyclin D2 (CCND2) (29), AKT serine/threonine kinase 1 (AKT1) (30), and GATA binding protein 3 (GATA3) (31) (Figure 7A). XLOC_011623 was co-expressed with 36 DEGs, which included CCND2 (29), AKT1 (30), GATA3 (31), and the MYC proto-oncogene (MYC) (32) (Figure 7B). CCND2 (29) and MYC (32) were included in 28 DEGs that co-expressed with CTD-3094K11.2 (Figure 7C). GATA3 (31) was one of 29 DEGs that co-expressed with AK027541 (Figure 7D). Fifteen DEGs were co-expressed with AF001548.5, including MYC (32) and high mobility group AT-hook 1 (HMGA1) (33) (Figure 7E). A total of 47 DEGs were co-expressed with RP1-278C19.3, including CCND2 (29), AKT1 (30), GATA3 (31), and MYC (32) (Figure 7F).

### HIF1A-AS2 Suppression Inhibits HASMC Proliferation

**Figure 8:**
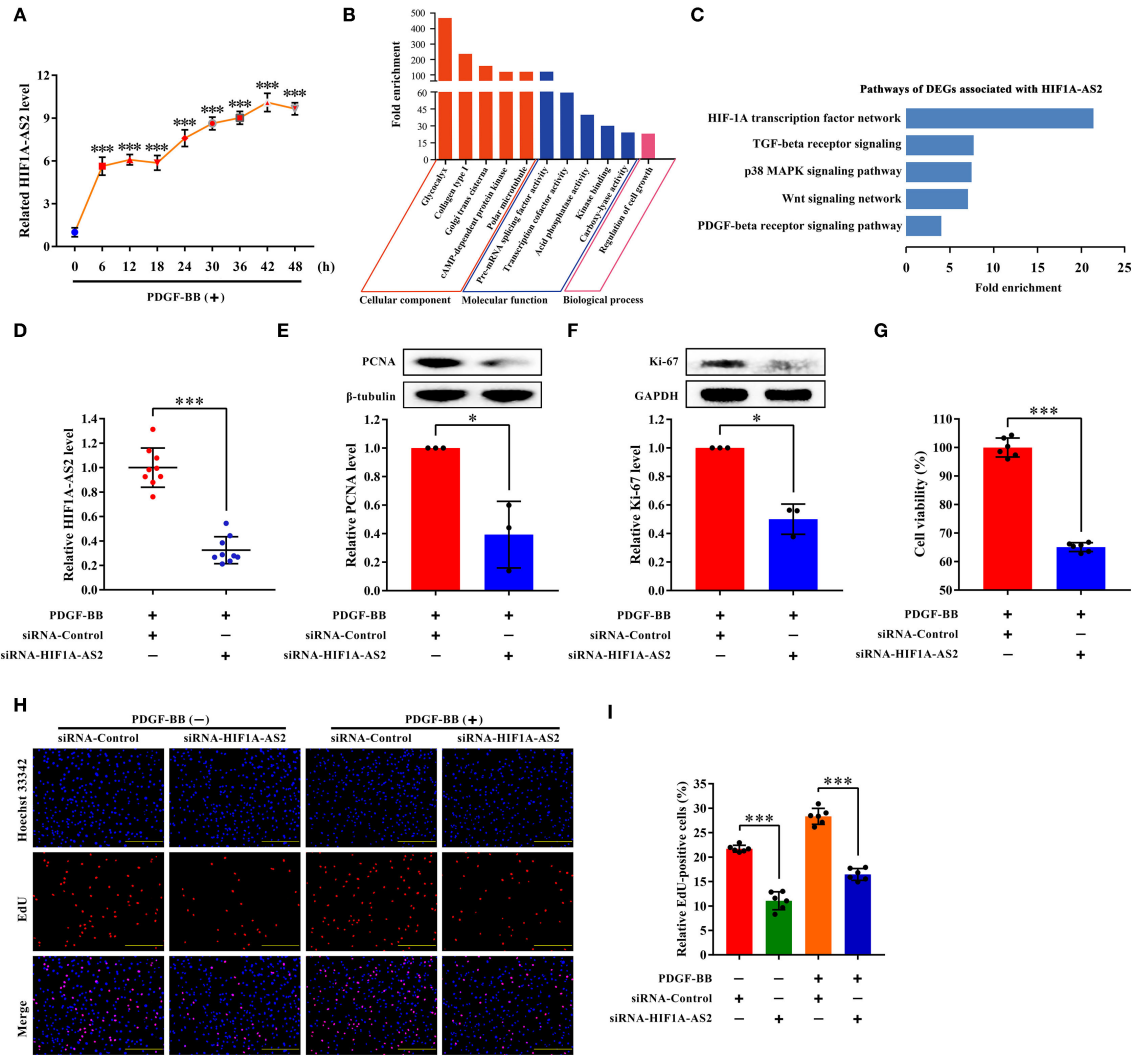
HIF1A-AS2 suppression inhibits HASMC proliferation. **(A)** qRT-PCR analysis of time-gradient changes of HIF1A-AS2 expression in HASMCs exposed to 10 ng/mL PDGF-BB treatment; normalized by β-actin. Data are presented as mean ± SD of three independent experiments, ***p < 0.001 vs. 0 h group. **(B)** GO analysis of 42 DEGs co-expressed with HIF1A-AS2. **(C)** Pathway analysis of 42 DEGs co-expressed with HIF1A-AS2. **(D)** qRT-PCR analysis of HIF1A-AS2 expression in HASMCs transfected with control or HIF1A-AS2 siRNA, stimulated with 10 ng/mL PDGF-BB; normalized by β-actin. Data are shown as mean ± SD of three independent experiments, ***p < 0.001 vs. siRNA-Control group. **(E,F)** Western blot analysis of PCNA and Ki-67 in HASMCs transfected with control or HIF1A-AS2 siRNA, stimulated with 10 ng/mL PDGF-BB; β-tubulin and GAPDH were utilized as a control, respectively. For each blot, bands were quantified and normalized toward the signal obtained for HASMCs transfected with control siRNA. Data are shown as mean ± SD of three independent experiments, *p < 0.05 vs. siRNA-Control group. **(G)** Function of HIF1A-AS2 on HASMC viability using CCK-8 assay after downregulating HIF1A-AS2 and stimulated with 10 ng/mL PDGF-BB. Data are shown as mean ± SD of three independent experiments, ***p < 0.001 vs. siRNA-Control group. **(H)** Effects of control or HIF1A-AS2 siRNA on DNA synthesis of HASMCs with or without 10 ng/mL PDGF-BB induction were determined using EdU incorporation assay. Blue fluorescence (Hoechst 33342) stood for cell nuclei and red fluorescence (EdU) showed HASMCs with DNA synthesis. The scale bar is 10 μm. **(I)** Relative EdU positive HASMCs. Data were expressed as the proportion of EdU positive HASMCs in total HASMCs. Data are shown as mean ± SD of three independent experiments, ***p < 0.001 vs. siRNA-Control group.

We selected HIF1A-AS2 to demonstrate its function in the regulation of HASMC proliferation based on the following reasons: (1) HIF1A-AS2 was up-regulated in proliferative HASMCs using microarray and qRT-PCR (Figures 2A, 8A). (2) Venn analysis of DELs with fold change ≥4 (*p* < 0.05) in proliferative HASMCs and DELs with fold change ≥2 (*p* < 0.05) in human advanced atherosclerotic plaques (GSE97210) (34) revealed three lncRNAs (HIF1A-AS2, RP11-841O20.2, and AC018647.3) were shared, in which HIF1A-AS2 is significantly augmented in peripheral blood monocyte cells of coronary artery disease patients (35), indicating that HIF1A-AS2 might be a promising therapeutic and diagnostic target for cardiovascular diseases. (3) Our data showed that HIF1A-AS2 might sponge 187 miRNAs (Figure 6A), five of which inhibit VSMC proliferation (5, 18, 19, 21–23). (4) GO and pathway analyses showed that 42 DEGs co-expressed with HIF1A-AS2 (Figure 7A) were enriched in cell proliferation-associated GO terms/pathways, such as regulation of cell growth (Figure 8B), HIF-1A transcription factor network, Wnt signaling, TGF-beta receptor signaling, p38 MAPK signaling, and PDGF-beta receptor signaling pathway (Figure 8C).

Small interfering RNA (siRNA) specifically suppressed approximately 70% of HIF1A-AS2 levels (Figure 8D). HIF1A-AS2 suppression reduced protein levels of proliferating cell nuclear antigen (PCNA) (Figure 8E) and marker of proliferation Ki-67 (Ki-67) (Figure 8F) in proliferative HASMCs. HIF1A-AS2 suppression decreased HASMC viability by 35%, as confirmed in the Cell Counting Kit-8 (CCK-8) assay (Figure 8G). HIF1A-AS2 knockdown inhibited HASMC proliferation by 49% in the absence of PDGF-BB stimulation and by 42% in the presence of PDGF-BB stimulation, as confirmed by the EdU incorporation assay (Figures 8H,I). These confirm that HIF1A-AS2 is a factor promoting HASMC proliferation.

### HIF1A-AS2 Restraint Inhibits HASMC Proliferation via miR-30e-5p/CCND2 Axis

**Figure 9:**
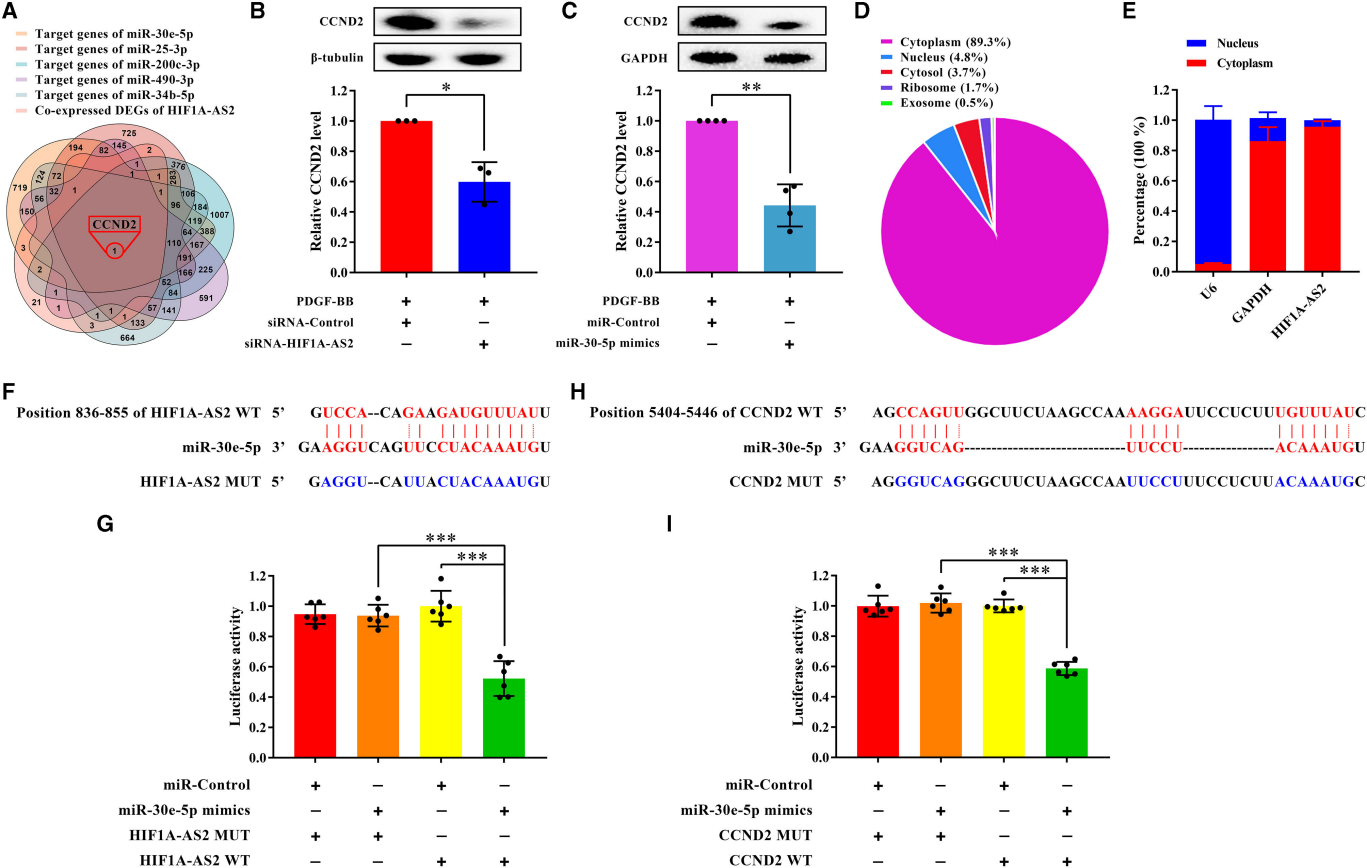
HIF1A-AS2 restraint inhibits HASMC proliferation via the miR-30e-5p/CCND2 axis. **(A)** Venn diagram revealed unique one shared DEG between potential target genes of miR-30e-5p/miR-25-3p/miR-200c-3p/miR-490-3p/miR-34b-5p and DEGs co-expressed with HIF1A-AS2. **(B,C)** Western blot analysis of CCND2 in HASMCs transfected with control or HIF1A-AS2 siRNA **(B)** and transfected with control or miR-30e-5p mimics **(C)** under 10 ng/mL PDGF-BB treatment; β-tubulin and GAPDH were utilized as a control, respectively. Data are shown as mean ± SD of three independent experiments, *p < 0.05 vs. siRNA-Control or miRNA-Control group. **(D)** HIF1A-AS2 subcellular localization prediction utilizing the LncLocator database. **(E)** HIF1A-AS2 was abundant in the cytoplasm of HASMCs. U6 and GAPDH acted as positive controls in the nucleus and cytoplasm, respectively. Data are shown as mean ± SD of three independent experiments. **(F)** Binding sites of HIF1A-AS2 and miR-30e-5p were predicted by the RNAhybrid database. **(G)** Confirmation of miR-30e-5p as a sponged target of HIF1A-AS2 using the dual-luciferase reporter gene assay. Data are presented as mean ± SD of three independent experiments, ***p < 0.001. **(H)** Sequence of the 3′-UTR of CCND2 mRNA that matches the miR-30e-5p seed sequence was predicted by the RNAhybrid database. **(I)** Verification of CCND2 as a target gene of miR-30e-5p utilizing the dual-luciferase reporter gene assay. WT: wild-type; MUT: mutant. Data are shown as mean ± SD of three independent experiments, ***p < 0.001.

We selected miR-30e-5p/CCND2 axis to validate whether it was regulated by HIF1A-AS2 based on the following reasons: (1) In the HIF1A-AS2/miRNAs interaction network (Figure 6A), five miRNAs [miR-30e-5p (18–20), miR-200c-3p (5), miR-25-3p (21), miR-490-3p (22), and miR-34b-5p (23)] suppress VSMC proliferation, in which miR-30e-5p is significantly down-regulated in coronary sinus blood in patients with heart failure (36). (2) Venn analysis between the target genes of these five miRNAs predicted by starBase (37) and the co-expressed DEGs of HIF1A-AS2 (Figure 7A) revealed unique one overlapped up-regulated co-expressed DEG, CCND2 (Figure 9A).

HIF1A-AS2 suppression or miR-30e-5p overexpression impaired CCND2 protein level in proliferative HASMCs (Figures 9B,C). LncLocator (38) (a lncRNA subcellular localization predictor) and nuclear/cytoplasmic RNA detection uncovered that HIF1A-AS2 was predominantly localized in the cytoplasm of HASMCs (Figures 9D,E). Therefore, we investigated whether HIF1A-AS2 acted as miR-30e-5p sponge to trigger positive regulation on CCND2. Using RNAhybrid (39), we found a potential binding site between HIF1A-AS2 and miR-30e-5p (Figure 9F). Dual-luciferase reporter gene assay elucidated that miR-30e-5p mimics restrained the luciferase activity of the HIF1A-AS2 wild-type (WT) plasmid, but not the mutant (MUT) plasmid (Figure 9G), suggesting that HIF1A-AS2 was a sponge for miR-30e-5p. In addition, a potential binding region between miR-30e-5p and CCND2 was also revealed by RNAhybrid (39) (Figure 9H). Dual-luciferase reporter gene assay uncovered that miR-30e-5p mimics inhibited the fluorescence activity of CCND2-WT plasmid, but did not affect the MUT plasmid (Figure 9I), indicating that CCND2 was a target gene of miR-30e-5p. These elucidate that HIF1A-AS2 promotes HASMC proliferation is, at least in part, triggered by the miR-30e-5p/CCND2 axis.

## Discussion

In this study, we investigated the function of lncRNAs in HASMC proliferation stimulated by the growth factor PDGF-BB. LncRNAs can *cis* regulate the levels of their neighboring genes (40, 41). Our GO and pathway analyses showed that DEGs neighboring DELs were enriched in the RNA-induced silencing complex (RISC; Figure 5D) that is required for miRNAs binding to target genes. LncRNAs have been identified to exert as ceRNAs to sponge miRNAs and regulate RISC, thus regulating VSMC proliferation, differentiation, and apoptosis (42–44). Hence, DELs might target VSMC proliferation-related genes by sponging miRNAs, regulating RISC, and *cis* regulating neighboring DEGs.

In addition, DEGs neighboring DELs were enriched in PDGF receptor binding associated-MF (Figure 5E) and TGF-beta receptor binding associated-MF (Figure 5F). Both PDGF (45, 46) and TGF-beta (47) signaling pathways function in VSMC proliferation. Another pathway enriched was the regulation of actin cytoskeleton (Figure 5H). Actin cytoskeleton remodeling is necessary for VSMC phenotypic switch (48, 49). Our group previously demonstrates that smooth muscle 22 alpha (SM22α), an actin-binding protein, participates in the organization of actin cytoskeleton in differentiated VSMCs by inducing F-actin bundling, thereby increasing VSMC contractility and mobility, and ultimately maintaining the differentiated phenotype of VSMCs (48). Over-expression of SM22α inhibits VSMC proliferation and neointima hyperplasia via reducing the response to mitogen stimuli (49). Thus, DELs might regulate VSMC proliferation by targeting the cytoskeleton-associated proteins, and by *cis* regulating their neighboring DEGs.

Analysis of DELs-miRNAs interaction networks (Figure 6) indicated that HIF1A-AS2 might sponge miR-30e-5p (18–20), miR-25-3p (21), miR-200c-3p (5), miR-490-3p (22), and miR-34b-5p (23) (Figure 6A). MiR-30e-5p (namely, miR-30e) targets and restrains insulin-like growth factor 2 (IGF2) (18), ubiquitin-conjugating enzyme E2 I (UBE2I) (19), and Ca^2+^/calmodulin-dependent protein kinase IIδ (CaMKIIδ) (20), thus diminishing VSMC proliferation (18–20), migration (19, 20), and dedifferentiation (18, 19). MiR-25-3p (namely, miR-25) targets and down-regulates cyclin-dependent kinase 6 (CDK6), triggering VSMC proliferation suppression (21). Upon PDGF-BB treatment, SUMO-conjugating enzyme Ubc9 interacts with and promotes the SUMOylation of Krüppel-like transcription factor 4 (KLF4), allowing the recruitment of transcriptional corepressors to the miR-200c-3p (namely, miR-200c) promoter to inhibit miR-200c-3p levels, leading to increased expression of target genes Ubc9 and KLF4, which further inhibits miR-200c-3p levels and ultimately promotes VSMC proliferation (5). In oxidized low-density lipoprotein-induced VSMC proliferation, miR-490-3p is down-regulated, while its target gene, pregnancy-associated plasma protein A (PAPP-A), is up-regulated, resulting in increased proteolysis of the PAPP-A substrate, IGF-binding protein-4 (IGFBP-4) (22). MiR-34b-5p mitigates VSMC proliferation by downregulating its target gene alpha-1 antitrypsin (AAT) (23). AC016747.3 might sponge miR-342-3p (Figure 6B), and miR-342-3p effectively attenuates PDGF-BB-induced VSMC migration and proliferation and overcomes endothelial cell inflammation (24). RP11-184I16.2 might sponge miR-128-3p, a miRNA that downregulates the expression of the target gene KLF4, and the inhibition of KLF4 levels promotes the expression of key VSMC gene MYH11 by mediating the methylation of the MYH11 promoter, thereby reducing VSMC proliferation and migration (25). Our results indicated that AC109586.1 might sponge miR-182-3p and miR-424-5p (Figure 6D). MiR-182-3p inhibits the asymmetric dimethylarginine-induced dedifferentiation, proliferation, and migration of VSMCs (26). MiR-424-5p (namely, miR-424) and its rat homologous gene miR-322 down-regulate the levels of target genes cyclin D1 and calumenin, leading to inhibition of VSMC proliferation and migration (27). Therefore, the upregulated HIF1A-AS2, AC016747.3, RP11-184I16.2, and AC109586.1 in proliferative HASMCs might act as ceRNAs to block the inhibition of VSMC proliferation by the corresponding miRNAs, thus promoting HASMC proliferation. In addition, miR-433-3p, which is significantly downregulated in myostatin-induced VSMC proliferation inhibition phenotype (28), might be sponged by RP11-504G3.1 (Figure 6E). MiR-494-3p, which was likely to be sponged by RP11-63E5.8 (Figure 6F), is also significantly downregulated in myostatin-induced VSMC proliferation inhibition phenotype (28). Therefore, the downregulated RP11-504G3.1 and RP11-63E5.8 in proliferative HASMCs might act as ceRNAs to block the promotion of VSMC proliferation by the corresponding miRNAs, thereby inhibiting HASMC proliferation.

Co-expression analysis of DELs-DEGs (Figure 7) indicated that CCND2, which promotes proliferation of pulmonary artery smooth muscle cells (29), might be a target of HIF1A-AS2, XLOC_011623, CTD-3094K11.2, and RP1-278C19.3. AKT1, which promotes VSMC proliferation (30), was a likely target of HIF1A-AS2, XLOC_011623, and RP1-278C19.3. GATA3, which suppresses vascular endothelial growth factor (VEGF) expression (31), was a likely target of HIF1A-AS2, XLOC_011623, RP1-278C19.3, and AK027541. XLOC_011623, CTD-3094K11.2, AF001548.5, and RP1-278C19.3 might target and upregulate the proto-oncogene MYC; MYC promotes VSMC proliferation by up-regulating DNA methyltransferase 1 (DNMT1) and inhibiting mitofusin 2 (MFN2) (32). HMGA1, one of the targets of AF001548.5, also promotes VSMC proliferation (33).

DELs-DEGs co-expression networks analysis demonstrated that lncRNA HIF1A-AS2, derived from the 3' end of hypoxia inducible factor 1 subunit alpha (HIF-1α) gene, was co-expressed with 42 DEGs in proliferative HASMCs (Figure 7A). Some of these DEGs were enriched in cell proliferation-related GO terms/pathways, such as regulation of cell growth (Figure 8B), HIF-1A transcription factor network, Wnt signaling, TGF-beta receptor signaling, p38 MAPK signaling, and PDGF-beta receptor signaling pathway (Figure 8C), suggesting that HIF1A-AS2 might regulate HASMC proliferation. Consistently, HIF1A-AS2 suppression effectively inhibited the phosphorylation levels of MAPK key components [i.e., ERK1/2 (extracellular signal-regulated protein kinase 1/2), p38 (p38 MAPK), and JNK (c-Jun N-terminal kinase)] (Supplementary Figures 1A,B), suggesting that HIF1A-AS2 was a potential HASMC proliferation activating factor. HIF1A-AS2 is overexpressed in multiple cancers (50–54), human umbilical vein endothelial cells (HUVECs) (55), and in peripheral blood monocytes from patients with cardiovascular disease (35). A natural antisense transcript named aHIF, a part of HIF1A-AS2, is specifically overexpressed in non-papillary kidney cancer (56), but can be also detected in normal tissues (57). HIF1A-AS2 functions by acting as ceRNA (53–55), or interacting with RBPs (58). In colorectal cancer, HIF1A-AS2 competitively sponges miR-129-5p to upregulate DNA methyltransferase 3 alpha (DNMT3A) (53). In breast cancer cells, HIF1A-AS2 activates the HIF-1α/VEGF pathway by competitively binding to miR-548c-3p (54). HIF1A-AS2 acts as a sponge for miR-153-3p and activates the HIF-1α/VEGFA/notch receptor 1 (Notch1) cascade, thus promoting viability, migration, and tube formation of HUVECs (55). Furthermore, HIF1A-AS2 directly binds to insulin-like growth factor 2 mRNA binding protein 2 (IGF2BP2)/ATP-dependent RNA helicase A (DHX9) proteins, resulting in increased expression of HMGA1, formation of glioblastoma stem-like cells, and adaptation to hypoxia in the tumor microenvironment (58).

Herein, we revealed that HIF1A-AS2 elevated HASMC proliferation (Figure 8), and HIF1A-AS2 suppression inhibited expression of CCND2 (Figure 9B), one of the co-expressed DEGs of HIF1A-AS2 (Figure 7A). Activation of HIF1A-AS2 on the proliferation of HASMCs might be achieved through positive regulation of CCND2 level. Based on the ceRNA function of HIF1A-AS2 (53–55), we constructed HIF1A-AS2/miRNAs interaction network (Figure 6A), in which miR-30e-5p eliminates VSMC proliferation (18–20), migration (19, 20), and dedifferentiation (18, 19) by targeting and impairing IGF2 (18), UBE2I (19), and CaMKIIδ (20). We elucidated that HIF1A-AS2 was a sponge for miR-30e-5p that targeted and diminished the CCND2 level (Figures 9A–I). Hence, we emphasized in mechanism that cytoplasmic HIF1A-AS2 promoted HASMC proliferation through the miR-30e-5p/CCND2 mRNA axis partially (Figure 9). In addition, HIF1A-AS2 upregulates target genes by binding to IGF2BP2/DHX9 protein (58), and IGF2BP2 protein upregulates CCND2 expression by binding to CCND2 mRNA (59), which indicates the potential of the HIF1A-AS2/IGF2BP2/CCND2 mRNA axis. However, the hypotheses need to be validated further in the future. Interestingly, HIF1A-AS2 suppression diminished protein levels of matrix metallopeptidase 9 (MMP9) and matrix metallopeptidase 2 (MMP2) in proliferative HASMCs (Supplementary Figures 1C,D), while elevated α-SMA (actin alpha 2, smooth muscle) protein level (Supplementary Figure 1E), suggesting that HIF1A-AS2 might also thrive HASMC migration and dedifferentiation, which needs further exploration in the future. Taken together, we document that HIF1A-AS2 promotes HASMC proliferation, at least in part, via the miR-30e-5p/CCND2 mRNA axis.

To our knowledge, this is the first report demonstrating that lncRNAs and mRNAs are differentially expressed in proliferative HASMCs induced by PDGF-BB. Furthermore, our results indicate that lncRNA HIF1A-AS2 promotes HASMC proliferation by the miR-30e-5p/CCND2 mRNA axis in some degree and highlight that HIF1A-AS2 might serve as a new therapeutic target for VSMC proliferative vascular diseases.

## Data Availability Statement

The datasets presented in this study can be found in online repositories. The names of the repository/repositories and accession number(s) can be found in the article/Supplementary Material.

## Author Contributions

S-GS and L-HD conceived and designed this study. J-JL, WC, MG, XX, and M-YD performed the bioinformatics analysis. J-JL, WC, MG, XX, M-YD, S-FW, L-YY, YW, K-XL, PK, BL, KL, and Y-ML performed the experiments. J-JL, WC, MG, and XX wrote the manuscript. All authors have read and agreed to the published version of the manuscript.

## Funding

This research was funded by the National Natural Science Foundation of China, Grant Numbers: 82170439, 81670273, and 81200215 to S-GS, Grant Number: 81670394 to L-HD; Natural Science Foundation of Hebei Province, Grant Numbers: H2021206399 and H2019206150 to S-GS.

## Conflict of Interest

The authors declare that the research was conducted in the absence of any commercial or financial relationships that could be construed as a potential conflict of interest.

## Publisher's Note

All claims expressed in this article are solely those of the authors and do not necessarily represent those of their affiliated organizations, or those of the publisher, the editors and the reviewers. Any product that may be evaluated in this article, or claim that may be made by its manufacturer, is not guaranteed or endorsed by the publisher.

## Supplementary Material

Supplementary Figure 1 Effects of HIF1A-AS2 suppression on MAPK signaling, migration, and differentiation in proliferative HASMCs. **(A–E)** Western blot analysis of p-ERK1/2, p-p38, p-JNK, MMP9, MMP2, and α-SMA in HASMCs transfected with control or HIF1A-AS2 siRNA, mediated by 10 ng/mL PDGF-BB; GAPDH was utilized as a control. Data are shown as mean ± SD of at least three independent experiments, ^*^*p* < 0.05, ^**^*p* < 0.01, ^***^*p* < 0.001 vs. siRNA-Control group.

Supplementary Table 1 Primers for qRT-PCR.

Supplementary Table 2 Differentially expressed lncRNAs.

Supplementary Table 3 Differentially expressed coding genes.
