# Supplementary figures and images for: Expression and Functional Analysis of lncRNAs Involved in Platelet-Derived Growth Factor-BB-Induced Proliferation of Human Aortic Smooth Muscle Cells
Source: Front Cardiovasc Med. 2021 Sep 7;8:702718. doi: 10.3389/fcvm.2021.702718 (PMC8452921; doi:10.3389/fcvm.2021.702718)

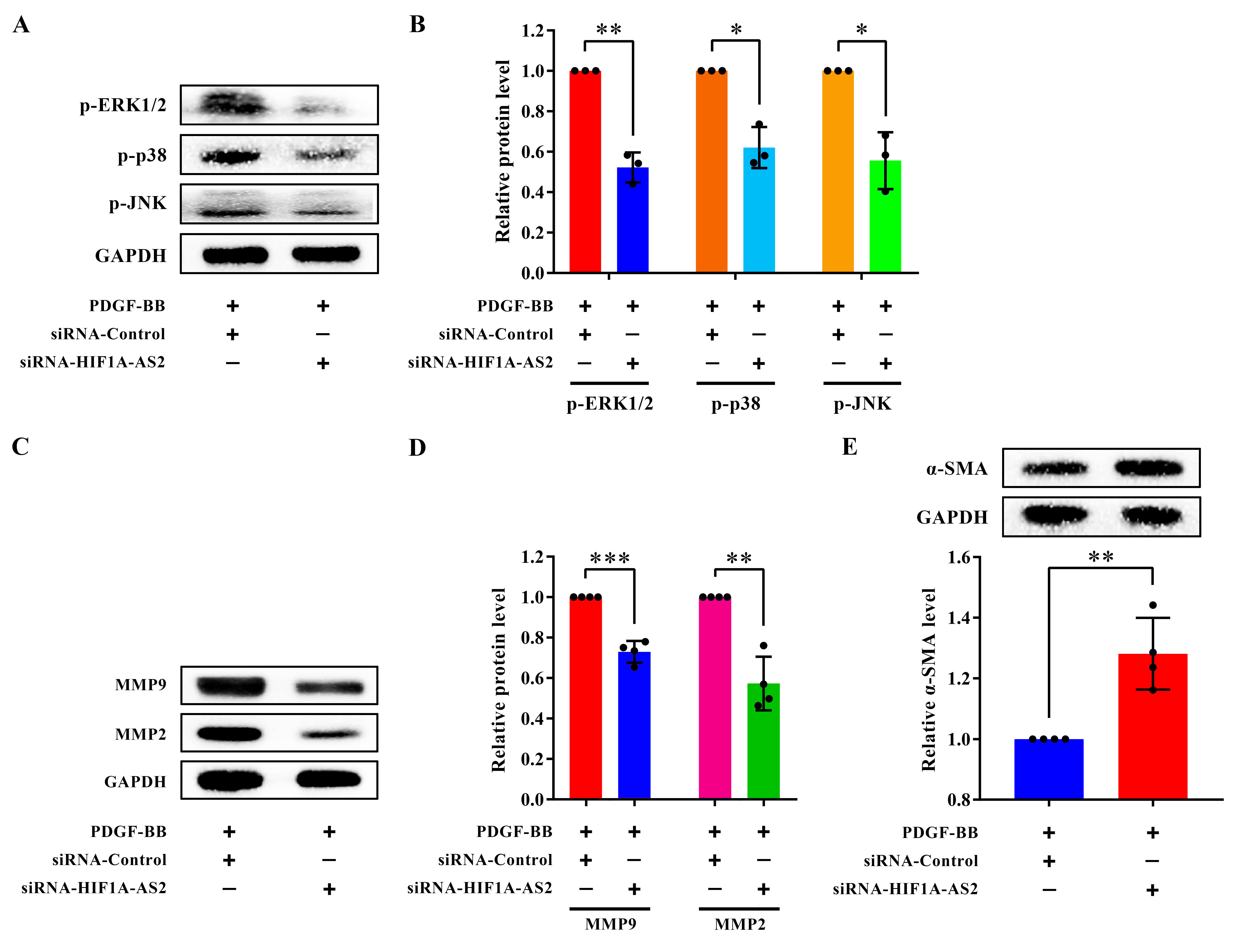

Supplement: Supplementary Figure 1 — Effects of HIF1A-AS2 suppression on MAPK signaling, migration, and differentiation in proliferative HASMCs. (A–E) Western blot analysis of p-ERK1/2, p-p38, p-JNK, MMP9, MMP2, and α-SMA in HASMCs transfected with control or HIF1A-AS2 siRNA, mediated by 10 ng/mL PDGF-BB; GAPDH was utilized as a control. Data are shown as mean ± SD of at least three independent experiments, *p < 0.05, **p < 0.01, ***p < 0.001 vs. siRNA-Control group. [file Image_1.tif]
