# Supplementary material for: Expression and Functional Analysis of lncRNAs Involved in Platelet-Derived Growth Factor-BB-Induced Proliferation of Human Aortic Smooth Muscle Cells
Source: Front Cardiovasc Med. 2021 Sep 7;8:702718. doi: 10.3389/fcvm.2021.702718 (PMC8452921; doi:10.3389/fcvm.2021.702718)
Supplement: Supplementary Table 1 — Primers for qRT-PCR. [file Data_Sheet_1.PDF]

Supplementary Table 1. Primers for qRT- PCR

RNAs	Primers sequence (5'to 3')
HIF1A-AS2	Forward：TTTTGGTCTGCCATCTATTACTTTT<br>Reverse：GAGTGAAGCAGTTCTCAGCATTA
CDKN2B-AS1	Forward：CTCCCCTATTCCCCTTATTTTATTC<br>Reverse：CTCGCTTTCCTTTCTTCCTTTTTC
LSM5	Forward：CTGCAGTGTTATAGAGCTTGTGG<br>Reverse：TCCTTCTTCCTTCTGGTGTGATT
FAM193A	Forward：AGAAGAAGGAGAGGCCAAGTAAAGAC<br>Reverse：CTGGATGGGGAGTGAGAAGAGGT
LINC00263	Forward：GCGCTCTGGCTTTTCCAT<br>Reverse：CCTCATAACCCGAGATTCTTTG
BC030753	Forward：CTGAGGGACGTGGATACAAAAAG<br>Reverse：ACTTTCTTCAGTGCAAGAGCAATG
RP11-552E20.1	Forward：GTGTCTGACATCTGCACTTTTTACT<br>Reverse：TCTAGCAATACCTGAGGCTGAGT
RP11-248N22.1	Forward：CCAGGATCGTGAATGTCTGAGA<br>Reverse：GAGAAACCGAACACTGATTTGATT
RP11-219B4.3	Forward：GGTTACCCTCAGGCCACAT<br>Reverse：AAGGACGGCGTTCTGGAT
CCNDBP1	Forward：GTTAGTGGCAGAGAATGGGAAGA<br>Reverse：GGTCAGGTGACACATAGGTGGATA
β-actin	Forward：GACTTAGTTGCGTTACACCCTTTCTTG<br>Reverse：ACTGCTGTCACCTTCACCGTTCC
GAPDH	Forward：TCGCTCTCTGCTCCTCCTGT<br>Reverse：GACTCCGACCTTCACCTTCC
U6	Forward：CGATACAGAGAAGATTAGCATGGC<br>Reverse：AACGCTTCACGAATTTGCGT

qRT-PCR: quantitative real-time polymerase chain reaction
